# Oncogenic roles and related mechanisms of the long non-coding RNA MINCR in human cancers

**DOI:** 10.3389/fcell.2023.1087337

**Published:** 2023-05-05

**Authors:** Ce Chao, Renzhe Tang, Jiamin Zhao, Dongmei Di, Yongxiang Qian, Bin Wang

**Affiliations:** ^1^ Department of Cardiothoracic Surgery, The Third Affiliated Hospital of Soochow University, Changzhou, China; ^2^ Department of Respiratory Medicine, The Third Affiliated Hospital of Soochow University, Changzhou, China

**Keywords:** lncRNA, MINCR, biomarker, treatment, human cancers

## Abstract

Long non-coding RNAs (lncRNAs) play vital roles in regulating epigenetic mechanisms and gene expression levels, and their dysregulation is closely associated with a variety of diseases such as cancer. Several studies have demonstrated that lncRNAs are dysregulated during tumor progression. Recently, the MYC-induced long non-coding RNA MINCR, a newly identified lncRNA, has been demonstrated to act as an oncogene in different cancers, including gallbladder cancer, hepatocellular cancer, colorectal cancer, non-small cell lung cancer, oral squamous cell carcinoma, nasopharyngeal cancer, and glioma. Moreover, MINCR has been reported to act as a biomarker in the prognosis of patients with different cancers. In this review, we summarize and analyze the oncogenic roles of MINCR in a variety of human cancers in terms of its clinical significance, biological functions, cellular activities, and regulatory mechanism. Our analysis of the literature suggests that MINCR has potential as a novel biomarker and therapeutic target in human cancers.

## Introduction

Cancers, as the leading cause of death around the world, has long been an area of great interest in research and medicine (Li et al., 2021). According to the latest cancer statistics of 185 countries, the incidence of different cancers is rising annually and the global cancer burden is expected to increase by 47% in 2040 compared with 2020. Relative to different cancers, the mortality rates of coronary heart disease and stroke have declined, and cancer development has been a barrier to increasing lifespans (Bray et al., 2021; Sung et al., 2021). Although there have been major therapeutic advancements in the past decade and some patients have benefited from gene therapy and immunotherapy, the overall survival rate has remained unchanged (Kruger et al., 2019; Cuciniello et al., 2021). Therefore, it is necessary to discover novel clinical biomarkers and therapeutic targets for different cancers.

Long non-coding RNAs (lncRNAs) are defined as a group of RNAs longer than 200 nucleotides in length without protein-coding functions (Chi et al., 2019). Due to their unique secondary and tertiary dimensional structures, lncRNAs have both RNA and protein functions (Novikova et al., 2013). LncRNAs have attracted wide attention, as they are a large group of molecules with multiple functions. In the human genome, only 2% of genes can encode proteins, and it has been estimated that the human genome contains approximately 56,946 lncRNAs, 2,700 miRNAs, and more than 20,000 protein-coding genes (Cagle et al., 2021). Moreover, lncRNAs can regulate the expression of protein-coding and protein non-coding genes through chromatin modification and remodeling, RNA splicing, and mRNA transcriptional and post-transcriptional regulation (Hu et al., 2021; Qian et al., 2021; Zhu et al., 2021). Some studies have reported that lncRNAs act as competing endogenous RNAs by binding and “sponging” miRNAs to regulate their target mRNAs. For example, the lncRNA KB-1460A1.5 competitively binds miR-130a-3p to regulate the expression of TSC1, which inhibits glioma progression (Xu L. et al., 2022). In addition, they can regulate the expression of genes in the nucleus and cytosol by establishing complexes with RNAs and proteins (Ma Z. et al., 2019). Several studies have revealed the potential of lncRNAs as clinical biomarkers and therapeutic targets for different cancers (Barik et al., 2021).

MYC-induced lncRNA (MINCR, ENSG00000253716), a novel lncRNA, was first identified and named by Doose et al. in an attempt to discover MYC-regulated lncRNAs potentially involved in lymphoma development (Doose et al., 2015). Since its identification and characterization, several studies have reported that MINCR can regulate multiple cancer phenomena, including cell proliferation, cell cycle regulation, apoptosis, migration, invasion, and the epithelial-to-mesenchymal transition (EMT) (Wang et al., 2016; Chen et al., 2019; Yu et al., 2020). In this review, we summarize the latest research on MINCR, as well as its aberrant expression, clinical significance, and regulatory mechanism in different cancers.

## Genetic information of MINCR

The *MINCR* gene is transcribed from the antisense chain of chromosome 8q24.3 that spans 5,943 bases of genomic DNA and is located intergenic to the coding genes *GLI4* and *ZNF696* with distances of 3 and 9.5 kb, respectively (Doose et al., 2015). In the latest assembly GRCh38. p14, MINCR (NC_000008.11) is located in chromosome 8 from 143,279,655 to 143,285,597 bases, a total of 5,943 bases. And, MINCR has two validated transcription variants, one containing three exons with 696 bases and one containing two exons with 395 bases. Which are identified as non-coding RNA. Doose et al. found that ENCODE annotates at least six different isoforms transcribed from the MINCR gene locus, with a long isoform (MINCR_L) composed of three exons and all others (MINCR_S, MINCR_S1, MINCR_S2, and MINCR_S3) containing two exons. And, MINCR were considered a lncRNA like lncRNA XIST by analyzing the coding potential calculator (CPC) score (Doose et al., 2015). Meanwhile, MINCR were preferentially enriched in the nuclear RNA fraction.

### MINCR in human cancers

**FIGURE 1:**
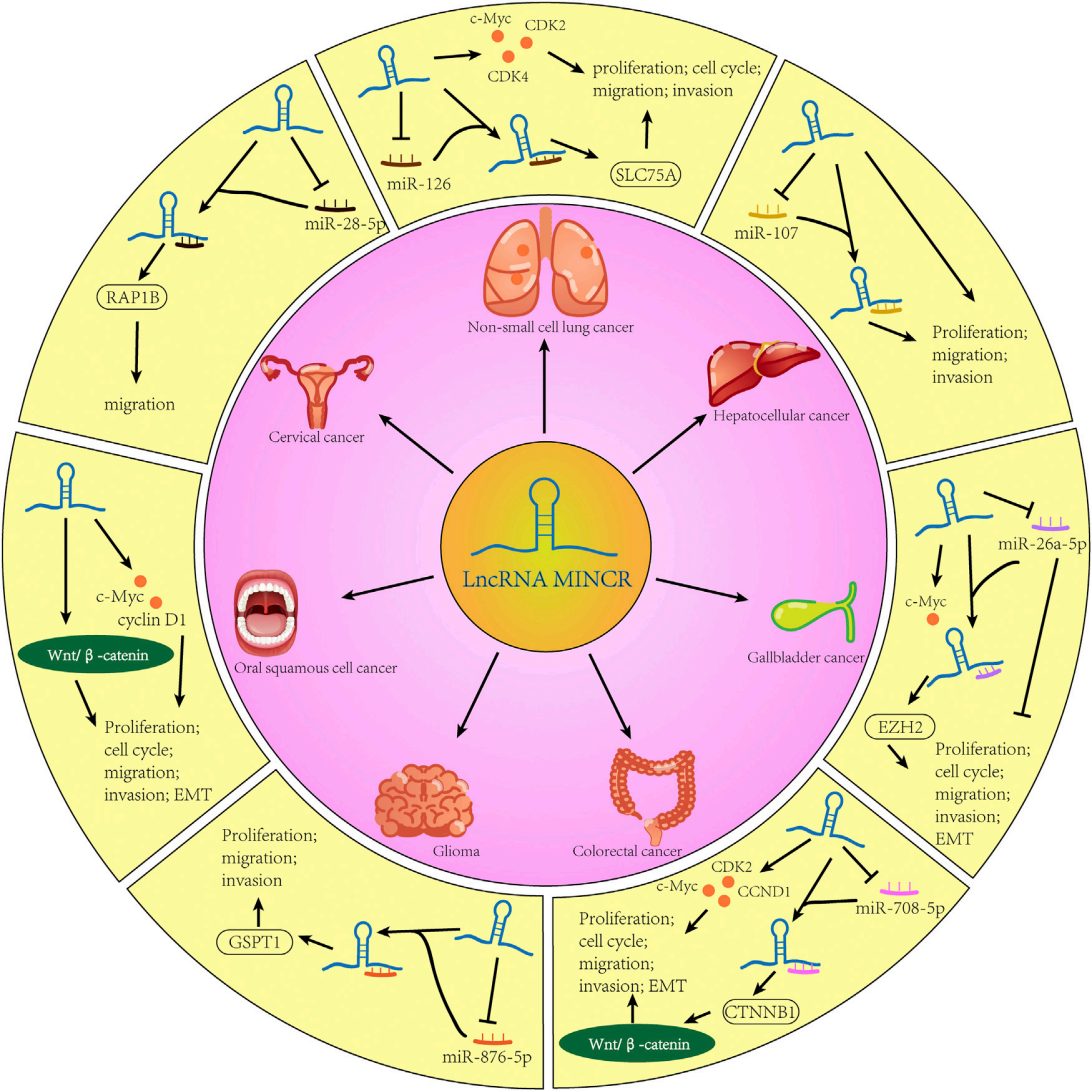
The function and regulating process of MINCR in multiple cancers.

**FIGURE 2:**
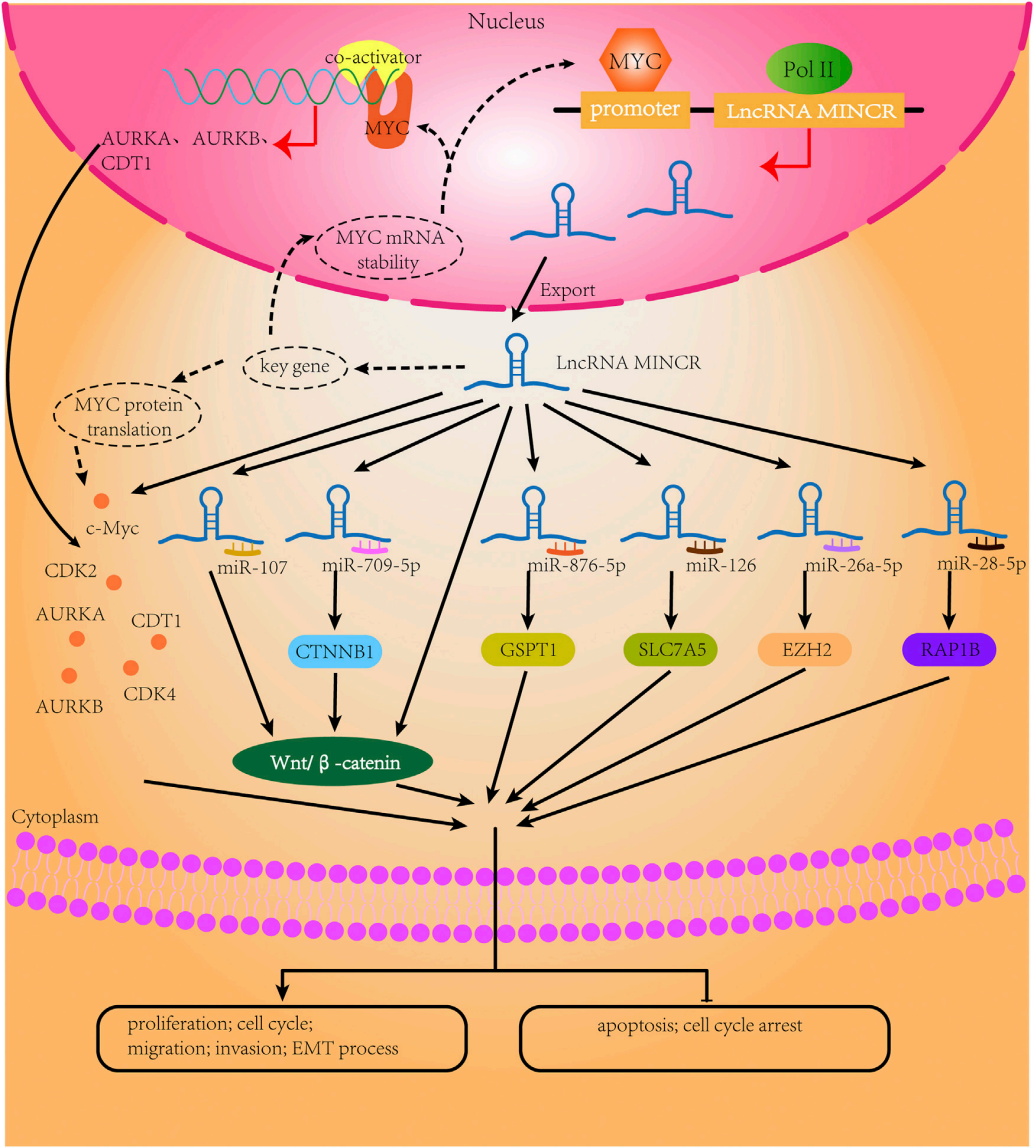
The biological mechanisms of MINCR in cancers. The content of the black broken circle represents the hypothetical biological mechanism.

The lncRNA MINCR has been demonstrated to be overexpressed in different cancers and to be significantly associated with the expression of the oncogene MYC (Pandini et al., 2021). MYC contributes to the development of many human cancers, and targeted therapies against MYC may be one of the most effective cancer treatments available today (Dang, 2012; Thng et al., 2021). MINCR also acts as an oncogene in multiple cancers. Recently, several studies have demonstrated that MINCR promotes clinicopathologic development by post-transcriptional gene regulation (Wang et al., 2016; Chen et al., 2019; Yu et al., 2020). However, at the time of this writing, there are only 22 articles in the PubMed database linked to the keywords “MINCR” and “cancer”. The clinical significance, functional characteristics, and regulatory mechanism of MINCR in different cancers are listed in Tables 1, 2 as well as Figures 1, 2.

**TABLE 1:** The expression, clinicopathological features, and clinical prognosis of MINCR in different human cancers.

Cancer type	Cases	Expression	Clinical characters	PMID
Non-small cell lung cancer	29	Overexpression	—	31481083
	35	Overexpression	OS	30528230
Hepatocellular cancer	70	Overexpression	—	29550632
	161	Overexpression	TNM stage; histological grade; OS	30556858
	75	Overexpression	TNM stage; lymph node metastasis; cirrhosis; OS	30947664
	52	Overexpression	OS	32521894
Gallbladder cancer	35	Overexpression	tumor sizes; lymph node metastasis; OS	27345740
Colorectal cancer	122	—	OS	30320902
Oral squamous cell cancer	80	Overexpression	lymph node metastasis; TNM stage; distant metastasis; OS	30777615

**TABLE 2:** The roles and functions of lncRNA MINCR in various cancers.

Cancer type	Role	Related molecule and pathway	Functions	Methods	PMID
Non-small cell lung cancer	Oncogenic	Cyclin A; cyclin D; CDK2; CDK4; c-Myc	Proliferation; cell cycle; apoptosis	*In vitro*	31481083
	Oncogenic	miR-126/SLC7A5	Proliferation; apoptosis; migration	*In vitro* and *in vivo*	30528230
Glioma	Oncogenic	miR-876-5p/GSPT1	Proliferation; apoptosis; migration; invasion	*In vitro* and *in vivo*	32333234
Hepatocellular cancer	Oncogenic	—	Proliferation; migration; invasion	*In vitro*	29550632
	Oncogenic	miR-107/β-catenin	Proliferation; apoptosis	*In vitro*	32521894
Gallbladder cancer	Oncogenic	c-Myc; miR-26a-5p/EZH2	Proliferation; cell cycle; apoptosis; migration; invasion; epithelial mesenchymal transformation	*In vitro* and *in vivo*	27345740
Colorectal cancer	Oncogenic	miR-708-5p/CTNNB1/Wnt/β-catenin	Proliferation; apoptosis; migration; invasion; epithelial mesenchymal transformation	*In vitro* and *in vivo*	32535381
Oral squamous cell carcinoma	Oncogenic	Wnt/β-catenin	Proliferation; apoptosis; migration; invasion; epithelial mesenchymal transformation	*In vitro*	30777615
Osteosarcoma	Oncogenic	Wnt/β-catenin	Proliferation; apoptosis; migration	*In vitro* and *in vivo*	35767742
Cervical cancer	Oncogenic	miR-28-5p/RAP1B	Migration	*In vitro*	35632705

### Burkitt lymphoma (BL)

Burkitt lymphoma, a highly aggressive non-Hodgkin lymphoma of B cells, has characteristics of rapid progression and extranidal involvement (Crombie and LaCasce, 2021). In the initial study, MYC is identified as the driving factor of BL (Bisso et al., 2019). Understanding the regulatory mechanism of MYC in BL is also an important direction for the diagnosis, treatment, and management of BL (Ott et al., 2013). MINCR was initially identified as a MYC-induced lncRNA in MYC-positive BL cells. In BL cells, MYC directly targets the promoter of MINCR, and MINCR regulates MYC recruitment to MYC-binding sites of some cell cycle-related genes, including *AURKA*, *AURKB*, *CDK2*, and *NCAPD2* (Doose et al., 2015). Therefore, targeting MINCR is an effective therapeutic approach in B cells with MYC positive.

### Non-small cell lung cancer (NSCLC)

As the second most common type of cancer worldwide, after breast cancer in females, lung cancer reportedly has the highest mortality rate (Sung et al., 2021). Non-small cell lung cancer is the main subtype, and small cell lung cancer is the other subtype. Based on The Cancer Genome Atlas database, MINCR has been demonstrated to be expressed higher in lung adenocarcinoma (LUAD) and lung squamous carcinoma (LUSC) tissues than in normal tissues (Chen et al., 2019). These results have been confirmed in two Chinese populations, namely, 29 and 35 NSCLC patients, and high MINCR expression is associated with poor prognosis (Chen et al., 2019; Wang et al., 2019). MINCR is involved in cancer development by regulating cell cycle-related genes such as *AURKA*, *AURKB*, and *CDK2* (Doose et al., 2015). Similarly, MINCR promotes lung cancer cell proliferation by enhancing the expression of c-Myc and its downstream effectors, including CDK2, CDK4, cyclin A, and cyclin D (Chen et al., 2019). Moreover, the inhibition of MINCR has been demonstrated not only to suppress migration and invasion but also to promote apoptosis by sponging miR-126 to decrease SLC7A5 expression (Wang et al., 2019). These findings show that MINCR not only regulates MYC transcription but also acts as an endogenous competing RNA to regulate SLC7A5.

### Glioma

Glioma is the most common and aggressive cancer of the central nervous system (CNS), accounting for 30%–40% of cases (Saxena and Jha, 2017; Zhao et al., 2021). Although there have been many therapeutic advancements in different cancers of the CNS, the prognosis of glioma is still poor with the mortality rate increasing steadily and patients with the disease living less than 2 years (Kim et al., 2021; Xu et al., 2021). Several studies have reported that numerous lncRNAs are aberrantly expressed in glioma. In addition, lncRNAs may act as clinical biomarkers and therapeutic targets (Li et al., 2018; Kim et al., 2021). Li et al. (Li Z. et al., 2020) demonstrated that MINCR expression is higher in glioma cell lines than in normal glial cells. And, the knockdown of MINCR expression inhibits cell proliferation, migration, and invasion and promotes apoptosis of glioma cell lines. Furthermore, MINCR negatively regulates and sponges miR-876-5p to control GSPT1 expression in glioma tissues and cell lines (Li Z. et al., 2020). Taken collectively, these findings indicate that the MINCR/miR-876-5p/GSPT1 axis plays a vital role in glioma development and progression.

### Hepatocellular carcinoma (HCC)

Hepatocellular carcinoma (HCC), the most common type of primary liver cancer, is characterized by high vascularization and recrudescence (Mossenta et al., 2020; Wong and Wong, 2021). Approximately 80% of cases are related to viral hepatitis, while the other causes are genetic predisposition, dietary exposure to aflatoxin, excessive alcohol consumption, non-alcoholic fatty liver disease, and liver cirrhosis (Gomaa et al., 2008). Approximately 10%–20% patients are diagnosed at an early stage and recommended for surgery. However, the treatment window for surgery is short, and most patients undergo systemic treatments such as trans-arterial chemoembolization and chemotherapy (Liu J. et al., 2021). Several multi-tyrosine kinase inhibitors have been approved for the treatment of advanced HCC, but the results are not encouraging (Rimassa et al., 2019). Therefore, it is important to identify novel clinical biomarkers and therapeutic targets for HCC. After analyzing the expression of MINCR in 70 pairs of HCC tumor tissues and adjacent normal tissues, it was demonstrated that the MINCR level is elevated in tumor tissues (Cao et al., 2018), consistent with the results of other studies (Jin et al., 2018; Lian et al., 2019; Li H. et al., 2020). In addition, high MINCR expression is correlated with high TNM classification (III–IV), low histologic grade, lymph node metastasis, and cirrhosis (Jin et al., 2018; Lian et al., 2019). Patients with HCC characterized by high MINCR expression have poor prognoses and low 3-year or 5-year survival rates (Jin et al., 2018; Lian et al., 2019; Li H. et al., 2020). Other studies have demonstrated that the knockdown of MINCR expression inhibited cell proliferation, migration, and invasion of HCC cells. The potential mechanism may be that MINCR downregulates miR-107 expression and upregulates CDK2 and c-Myc expression (Lian et al., 2019; Li H. et al., 2020). The *β*-catenin signaling pathway, as a driver of HCC progression, has also been reported to be involved in the regulatory mechanism of MINCR (Li H. et al., 2020; Wang et al., 2021). In summary, MINCR acts as oncogene by regulating c-Myc and miR-107 in HCC.

### Gallbladder cancer (GBC)

Gallbladder cancer (GBC) is a rare and an aggressive malignancy. According to recent statistics, the incidence of GBC is significantly related to the geographical location. For example, most cases are concentrated in Latin America and Southeast Asia (Sharma et al., 2017; Okumura et al., 2021). Gallbladder cancer is difficult to diagnose at an early stage, and it is usually discovered accidentally during gallstone surgery or the assessment of symptoms of icterus due to blockage (Baiu and Visser, 2018). In the past two decades, the mortality rate of GBC has been decreasing steadily, although the 5-year survival rate is approximately 20%. The survival of patients who are not recommended for surgery remains at 2%–13% (Okumura et al., 2021). To improve the overall survival of patients with GBC, novel therapeutic targets need to be identified. Compared with adjacent normal gallbladder tissues, MINCR expression is significantly increased in GBC tissues. In addition, MINCR expression is higher in GBC patients with larger tumor sizes, more lymph node metastatic lesions, and shorter overall survival times than in those with smaller tumor sizes, fewer lymph node metastatic lesions, and longer overall survival times. A subsequent study confirmed that MINCR promotes cell proliferation and invasion. The knockdown of MINCR in GBC cells induces the arrest of the cell cycle at the G1/S stage and the mesenchymal-to-epithelial transition (Wang et al., 2016). In terms of the regulatory mechanism, MINCR upregulates the expression of EZH2 by sponging miR-26a, which further enhances the MYC/miRNA/EZH2 axis (Zhao et al., 2013; Wang et al., 2016). After injecting NOZ cells of nude mice with the MINCR small interfering RNA, we observed that tumor growth and EZH2 expression were inhibited (Wang et al., 2016). Taken collectively, these findings indicate that MINCR is a potential therapeutic target for clinical intervention.

### Colorectal cancer (CRC)

Colorectal cancer (CRC) is a common malignancy with high morbidity and mortality rates. The incidence of CRC is increasing in individuals younger than 50 years of age (Wieszczy et al., 2020; Collaborative et al., 2021; Swierczynski et al., 2021). After analyzing 122 cases from the Gene Expression Omnibus database, researchers have reported MINCR to have prognostic value for CRC patients (HR = 2.5187, *p* = 0.0099) (Zhang et al., 2019). Compared with a normal colon epithelial cell line (NCM460), MINCR expression is significantly increased in SW620, HCT116, RKO, and HT29 CRC cell lines. In terms of biological activities, cell proliferation, migration, invasion, and the EMT are inhibited after knockdown of MINCR expression in CRC cell lines. Furthermore, the Wnt/β-catenin pathway is involved in these biological processes. It has been reported that MINCR regulates the expression of *β*-catenin by competitively binding miR-708-5p, and these results have been verified by *in vivo* experiments (Yu et al., 2020). Taken collectively, MINCR may be a potential prognostic biomarker in CRC. However, further studies are needed to determine whether MINCR may be a potential diagnostic marker in this population.

### Head and neck cancer (HNC)

Head and neck cancer (HNC), which includes different cancers of the aerodigestive tract, is the seventh most common cancer (Mehanna et al., 2010). Its main risk factors include tobacco and alcohol use, as well as infection with human papillomavirus or Epstein-Barr virus (Mody et al., 2021). The treatment of this disease has a great impact on patients and their quality of life, so it is important to effectively manage HNC and to improve patient life expectancy (Mody et al., 2021). Oral squamous cell cancer (OSCC) is the main type of HNC. In 80 pairs of OSCC tissues and matched normal tissues, MINCR expression has been demonstrated to be significantly increased in OSCC tissues. Moreover, MINCR expression is associated with the clinicopathological features of OSCC patients such as TMN stage, lymph node metastasis, and distant metastasis. In addition, the MINCR expression level can also guide the clinical prognosis of OSCC patients (Lyu et al., 2019). The knockdown of MINCR inhibits cell proliferation and invasion by inhibiting the Wnt/β-catenin pathway in OSCC, similar to other human cancers (Lyu et al., 2019; Li H. et al., 2020; Yu et al., 2020).

### Other types of cancer

Cervical cancer (CC), one of the most common gynecological tumors, is mainly related to persistent infection with high-risk human papillomavirus (HR-HPV). Human papillomavirus type 16 (HPV16) is the most frequent genotype of HPV (Singini et al., 2022). It has been reported that E6 oncoprotein is expressed by intra-typical variants of HPV16. Meanwhile, MINCR is overexpressed in the CC cell line C33-A after transfecting E6, which is negatively correlated with the expression of miR-28-5p and positively correlated with the expression of RAP1B. Bioinformatics analysis revealed the significance of the MINCR/miR-28-5p/RAP1B axis in CC (Perez-Bacho et al., 2022), although additional studies are needed to define its role in the disease.

Another bioinformatics study found that MINCR is a cuproptosis-associated lncRNA associating with poor prognosis in patients with clear cell renal cell carcinoma (Xu S. et al., 2022). Additional studies are needed to understand its regulatory mechanism in clear cell renal cell carcinoma.

In osteosarcoma, the knockdown of MINCR inhibited cell proliferation and migration and promoted apoptosis. Furthermore, c-Myc and cell cycle-related genes were downregulated after knockdown of MINCR expression in osteosarcoma cells (Bai et al., 2022).

### MINCR in other diseases

Furthermore, we have summarized the regulatory mechanisms of MINCR in other diseases such as osteoarthritis, neurodegeneration, and schizophrenia. In LPS-induced acute lung injury, MINCR was overexpressed in a LPS time- and dose-dependent manner and depression of MINCR could reduce inflammatory infiltration by miR-146b-5p/TRAF6 axis (Gao and Zhang, 2021). While MINCR was downregulated in osteoarthritis (OA) and IL-1β induced chondrocytes. Mechanistically, MINCR reduced the expression of extracellular matrix metalloproteinases and inhibited chondrocyte apoptosis through miR146a-5p/BMPR2 pathway in OA (Li et al., 2022). Therefore, MINCR paly different roles in different target cells. In the Amyotrophic Lateral Sclerosis, MINCR was downregulation while upregulation in glioma patients. MINCR may be involved in neurodegenerative diseases through ST8SIA1, DSG2, RET, or NEDD9 (Pandini et al., 2021). As we all known, some molecular, such as p53, cyclin D, cyclin E, cyclin F, Pin1, and so on, are either complementarily deregulated or share remarkably overlapping functional pathways between cancer and neurodegenerative diseases (Seo and Park, 2020). MINCR may be one of them. However, the detailed mechanism of MINCR still needs to be verified in the neurodegenerative diseases. Another study showed MINCR was identified as a vital gene in the lncRNA-miRNA network of schizophrenia. And, MINCR could be regarded as biomarker candidates for schizophrenia (Sabaie et al., 2021).

### Regulatory mechanisms of MINCR

#### Crosstalk between MINCR and MYC

The MYC regulatory network is one of the most frequently dysregulated networks in cancer. LncRNAs, as important regulators of gene expression networks, are involved in the MYC regulatory network, and the relationship between different lncRNAs is intricate (Iaccarino, 2017; Fatma and Siddique, 2020; Tu et al., 2021). MINCR is upregulated by MYC in MYC-positive B-cell lymphomas. Doose and colleagues (Doose et al., 2015) have reported that the relationship between MINCR and MYC goes beyond that of an expression correlation. An analysis of published MYC ChIP-seq data shows that MYC binds the MINCR promoter, which has been confirmed in several cell lines, including BL cell lines and the P493-6 cell line (Doose et al., 2015; Doose et al., 2016; Iaccarino, 2017). Furthermore, MINCR affects the transcriptional activity of MYC. The knockdown of MINCR can decrease the expression of c-MYC and MYC target genes (Wang et al., 2016; Cao et al., 2018; Chen et al., 2019). Moreover, MYC binds to the promoters of its target genes (Doose et al., 2015). Another study has reported that some lncRNAs can interact directly or indirectly with MYC (Tu et al., 2021). For example, lncRNA GLS-AS, which is suppressed by MYC after nutrient stress, can decrease MYC expression by impairing the glutaminase-mediated stabilization of MYC (Deng et al., 2019). Similarly, the MYC transcription-mediated repression of the lncRNA FGF13-AS1 can degrade the MYC mRNA by disrupting IGF2BP1 function (Ma F. et al., 2019). Therefore, we speculate that the knockdown of MINCR may inhibit some key genes that maintain the stability of the MYC mRNA or protein. MINCR can also recruit the MYC co-activator to the promoters of some genes and guide MYC binding to these genes, which may be a positive-feedback mechanism. Additional studies are needed to define the interactions between MINCR and MYC.

#### MicroRNA-mediated regulation

LncRNAs function as competing endogenous RNAs (ceRNAs) and sponging microRNAs (miRNAs) in a variety of diseases (Liu L. et al., 2021). The ceRNA hypothesis reveals the functions of lncRNAs and the relationships between coding RNAs and non-coding RNAs. LncRNAs bind miRNAs and sponge miRNA response elements to regulate the expression of mRNAs (Yang et al., 2020). Moreover, miRNAs regulate the stability of lncRNAs (Ballantyne et al., 2016). The lncRNA/miRNA/mRNA axis involves interactions among the three RNA types and reveals the regulatory mechanisms of multiple genes in different diseases. The miRNAs targeted directly by MINCR are miR-126, miR-876-5p, miR-107, miR-26a-5p, miR-708-5p, and miR-28-5p in different cancers. It is possible that MINCR functions with other non-coding RNAs to regulate the same miRNA in disease, thereby altering the targeted miRNAs in different cancers.

#### Oncogenic signaling pathways

The Wnt/β-catenin signaling pathways is classical oncogenic signaling pathways. Given that MYC is a target of the Wnt/β-catenin signaling pathway, MINCR is closely related to this highly conserved signaling pathway (Jahangiri et al., 2021). In cancer, the Wnt/β-catenin signaling pathway is aberrantly activated, which is associated with tumor progression and poor prognosis (Tewari et al., 2021; Yu et al., 2021; Hiremath et al., 2022). In general, Wnt proteins mediate extracellular signals that activate this pathway by binding to the membrane receptors Frizzled and LRP5/6. Once activated, the Wnt pathway stabilizes *β*-catenin, which translocates to the nucleus, thereby upregulating the expression levels of genes involved in cell proliferation, migration, invasion, and apoptosis (Liu et al., 2022). In colon cancer, the pattern of MINCR expression is similar to that of *β*-catenin (Yu et al., 2020). In multiple cancers, *β*-catenin-activating mutations are very common (Boyault et al., 2007; Jones et al., 2012), and in elderly patients with CRC, the elevated expression of nuclear *β*-catenin indicates poor prognosis (McCleary et al., 2016). Therefore, the role of MINCR in activating the Wnt/β-catenin signaling pathway by increasing the expression of *β*-catenin is of clinical significance. Moreover, MINCR can also regulate the Wnt/β-catenin signaling pathway by sponging miR-107 in hepatocellular cancer (Yu et al., 2020). It is possible that an activator of the Wnt/β-catenin pathway can partly reverse the suppressive effects of MINCR knockdown in OSCC (Lyu et al., 2019). These results suggest the Wnt/β-catenin signaling pathway is the important part of MINCR promoting cancer development. Of course, more signaling pathways that MINCR may be involved in needs to be further studied.

## Discussion

At present, more and more researchers have devoted themselves to exploring the role of non-coding RNAs in tumors, especially lncRNAs and circRNAs, because they are abundant *in vivo* and widely involved in the occurrence, development, and metastasis of tumors. So far, lncRNA MINCR has been considered to be an oncogene, like MYC. Therefore, MINCR is expected to become a brand-new budding star in the diagnosis, treatment and clinical application of tumors.

Clinically, MINCR was tightly relevant to TNM staging, tumor size, histological grade, and overall survival. In the beginning, Doose et al. reported that MINCR was related to cell proliferation and cell cycle (Doose et al., 2015). A growing number of experiments verified its proliferative effects *in vitro* and *in vivo*. Not only that, some studies also reported that MINCR has also been associated with lymph node metastasis and even distant metastasis. Consistent with this, MINCR has also been shown to promote tumor migration and invasion at the cellular level. Moreover, MINCR can also promote EMT progression of tumors, such as gallbladder cancer, colorectal cancer, and oral squamous cell carcinoma. Presumably, EMT process maybe change cell-cell adhesion, cellular extracellular matrix as well as cytoskeletal remodeling during cancer progression (Polyak and Weinberg, 2009). Therefore, MINCR may play a vital role in all stage of tumor. In the published articles, MINCR has been shown to be overexpressed in various tumors, whether adenocarcinoma (Wang et al., 2019) or squamous cell carcinoma (Lyu et al., 2019). However, there is no literature reporting on the potential of MINCR as a diagnostic biomarker. Clinically, chemotherapeutic drugs can also be divided into cell cycle nonspecific agents (CCNSA) and cell cycle specific agents (CCSA) according to their effects on cell cycle (Ocio et al., 2014). MINCR, as a gene related to cell cycle, may also be altered in response to chemotherapy. Meanwhile, EMT process is related to cisplatin resistance (Ashrafizadeh et al., 2020). Hence, more clinical roles of MINCR remain to be discovered.

Mechanically, MINCR acts an oncogene in cancers via regulating MYC, targeting miRNAs, and mediating Wnt/β-catenin signaling pathways. For example, MINCR upregulates the expression of EZH2 by sponging miR-26a, which further enhances the MYC/miRNA/EZH2 axis in gallbladder cancer (Zhao et al., 2013; Wang et al., 2016). More studies verified that MINCR affects the transcriptional activity of MYC and is positively correlated with the expression levels of c-myc. In cancer, the aberrant Wnt/β-catenin signaling pathways facilitates cancer stem cell renewal, cell proliferation and differentiation (Zhang and Wang, 2020). It is reported that MINCR mediates the Wnt/β-catenin signaling pathways by downregulating the expression level of *β*-catenin protein. While MINCR is also involved in the NF-κB signaling pathway by miR-146b-5p/TRAF6 axis in inflammation (Gao and Zhang, 2021). Although Yu et al. proved that Wnt/β-catenin signaling pathways are involved in MINCR-modulating tumors (Yu et al., 2020). There is no literature to verify that the Wnt/β-catenin signaling pathways is the main pathway of MINCR-modulating tumors. Meanwhile, recently studies have identified MINCR as a cuproptosis-related gene by bioinformation analysis in kidney renal clear cell carcinoma (Xu S. et al., 2022; Hong et al., 2022). While the detailed biological mechanism is unclear. Therefore, the regulatory networks of MINCR remain to be improved.

## Conclusion and future perspectives

Currently, our knowledge of MINCR is very limited, and this lncRNA has not been thoroughly explored in cancers. The lncRNA MINCR is involved in tumor progression and associated with poor prognosis. In particular, the role of MINCR in cancer cell proliferation makes it a promising target for the treatment of cancer, similar to MYC. In this review, we discussed the relationship between MINCR and MYC, as well as the function and the regulatory mechanism of MINCR in different cancers. Targeting MINCR not only interferes with the development of tumors but also directly suppresses the oncogene MYC. Therefore, interfering with the function of MINCR may improve cancer treatment and patient prognosis. Although MYC can directly target MINCR, the regulatory mechanism between the two molecules remains to be investigated. As a relatively new lncRNA, the functions of MINCR in other cancers, such as those of the breast and the blood, need to be investigated, and additional *in vivo* studies need to be performed in other cancers such as HCC and OSCC. The expression of MINCR is associated with the prognosis of patients with cancer, although it is unclear whether MINCR can be an effective diagnostic marker of the different tumor types. In terms of the regulatory mechanism, MINCR can directly target miRNAs to regulate multiple biological processes. However, there are few studies on RNA-binding proteins. Lastly, chemoradiotherapy resistance is a challenging bottleneck in the treatment of patients, and MYC is an important therapeutic target. Targeting MINCR in this population may result in new breakthroughs in the treatment of MINCR-expressing tumors.

In conclusion, MINCR has been demonstrated to function as oncogene in some human cancers by regulating the oncogene MYC, as well as different miRNAs, signaling pathways, and proteins. MINCR has potential as a novel clinical biomarker and therapeutic target in human cancers, which requires further validation.
